# Feasibility and Acceptability of a Real-Time Telerehabilitation Intervention for Children and Young Adults with Acquired Brain Injury During the COVID-19 Pandemic: An Experience Report

**DOI:** 10.5195/ijt.2021.6423

**Published:** 2021-12-16

**Authors:** Maria Chiara Oprandi, Alessandra Bardoni, Luisa Corno, Agata Marchetti Guerrini, Luigi Molatore, Luisella Negri, Elena Beretta, Federica Locatelli, Sandra Strazzer, Geraldina Poggi

**Affiliations:** 1 Scientific Institute, Irccs E. Medea, Neuro-Oncological and Neuropsychological Rehabilitation Unit, Bosisio Parini, Lecco, Italy; 2 Scientific Institute, Irccs E. Medea, Acquired Brain Injury Unit, Bosisio Parini, Lecco, Italy

**Keywords:** Acceptability, Acquired brain injury, Developmental age, Feasibility, Neuropsychology, Real-time, Speech therapy, Telerehabilitation

## Abstract

This study examined the feasibility and acceptability of a telerehabilitation intervention during the COVID-19 pandemic in a sample of children and young adults with Acquired Brain Injury (ABI). Thirteen patients and/or their families agreed to participate in the speech and neuropsychological telerehabilitation sessions. The treatment was synchronous, patient centered and aimed at improving specific abilities. Sessions were held twice a week over a 10-week period. Two questionnaires were completed both by parents and therapists to assess feasibility and acceptability. Neither technical issues nor clinical obstacles were found. The quality of the therapeutic relationship played a key role in the intervention. Synchronous telerehabilitation provided several advantages both for patients and therapists. Moreover, the patient centered intervention eased the burden of the caregivers at a time of high stress. The real-time telerehabilitation treatments were deemed suitable for children and young adults with ABI. Further studies are needed to support the use of telerehabilitation as an integral part of their standard care.

When the severe acute respiratory syndrome coronavirus 2 (SARS-CoV-2) or COVID-19 (WHO, 2020) spread from China to the rest of the world, rehabilitation centers had to stop all services to prevent the diffusion of the epidemic (Fazzi et al., 2020; Negrini et al., 2020a) and to protect both health care workers and patients. Across Europe, 87% of outpatient activities were suspended for 318,000 patients per day in Italy, Belgium, and the UK, for a total estimated 1.3 to 2.2 million patients (Negrini et al., 2020a). In addition, schools in Italy closed in February 2020 when the emergency began (Official Journal of the Italian Republic, 2020).

Because of the need to apply social distancing measures to protect the most vulnerable populations (Trabacca et al., 2020), children with disabilities such as Acquired Brain Injury (ABI), experienced several negative consequences during the pandemic due to their isolation (Ferreira Meireles & Ferreira de Meireles 2020). They were deprived, at least at the beginning of the pandemic, of their necessary therapies (Ferreira Meireles & Ferreira de Meireles, 2020) and the support of their teachers, doctors, therapists, educators (Trabacca et al., 2020) and peers during a time of great stress (Alexander, 2020). This led them to experience feelings of loneliness, symptoms of depression and anxiety (Ferreira Meireles & Ferreira de Meireles, 2020; Ren et al., 2020), and caused a regression in their abilities and a worsening of their social and school skills (United Nations, 2020).

Moreover, patients with ABI show an intrinsic frailty as a consequence of the disease, characterized by cognitive, behavioral, neuro- and psychological impairment (Corti et al., 2019).

Further complicating matters during the first phases of the COVID-19 pandemic was that caregivers of children with a disability were often asked to take the place of therapists and provide rehabilitation interventions in the home environment (Dhiman et al., 2020). This rendered these requests particularly challenging to achieve and increased caregiver stress and anxiety (Dhiman et al., 2020; Ren et al., 2020).

Health care professionals also went through a time of great stress because they were forced to reschedule their priorities and to change their standard working methods (López-Cabarcos et al., 2020).

Renowned Italian and international associations such as the Italian Society of Physical and Rehabilitation Medicine (2020), the Italian Society of Child and Adolescent Neuropsychiatry (2020), the American Psychological Association (2020), the American Telemedicine Association (2020), and the Inter Organizational Practice Committee (2020), strongly recommended protecting the most vulnerable categories of the population, by immediately reorganizing interventions to be held remotely, to guarantee continuity of care.

Indeed, telerehabilitation allows professionals to provide rehabilitation services to patients at a distance (Camden et al., 2019). Effective clinical and technological suggestions to use telerehabilitation in practice were made, before (American Psychiatric Association, 2018; American Telemedicine Association, 2017; Brennan et al., 2010) and also during the pandemic (American Telemedicine Association, 2020; Inter Organizational Practice Committee, 2020; Italian Society of Physical and Rehabilitation Medicine, 2020; Italian Society of Child and Adolescent Neuropsychiatry, 2020). These included the availability of telehealth devices in an institution (i.e., an appropriate virtual platform) (American Psychiatric Association, 2018; American Telemedicine Association, 2017, 2020; Inter Organizational Practice Committee, 2020; Italian Society of Child and Adolescent Neuropsychiatry, 2020). Therapists should have telehealth competency, be prepared to provide tests and rehabilitation materials remotely, and be able to modify these tests and materials, their educational techniques, and the setting as required (American Telemedicine Association, 2017, 2020; Brennan et al., 2010). Moreover, professionals need an available caregiver or a family member, when necessary to help the patient during the encounters: parents can help to create a quiet place for the telehealth session, turn on the devices and log into them for the child (American Psychological Association, 2020; American Telemedicine Association, 2017, 2020; Brennan et al., 2010; Inter Organizational Practice Committee, 2020). In this regard, it was suggested providing some extra time for relationship building between the therapist, the patients, and their caregivers (Koterba et al., 2020). Importantly, the telehealth sessions must be adapted to the patient's abilities (American Psychiatric Association, 2018; American Psychological Association, 2020; Brennan et al., 2020; Inter Organizational Practice Committee, 2020), especially considering the ABI sequelae on children (Corti et al., 2019).

Telerehabilitation can be delivered via different modalities (Coleman et al., 2015). Synchronous telerehabilitation is performed with audio and video interaction in real time, simulating traditional in-person therapy (American Speech-Language-Hearing Association, 2021; American Telemedicine Association, 2020; Coleman et al., 2015). On the other hand, asynchronous interventions take advantage of “store-and-forward” software to conserve and transmit images or data that need to be subsequently viewed or interpreted by the therapists. This can also be used to provide information and recommendations to caregivers who can access them at a later time (American Telemedicine Association, 2017, 2020; Brennan et al., 2020). Finally, hybrid telerehabilitation is a combination of the previous two modalities (American Speech-Language-Hearing Association, 2021; Coleman et al., 2015).

Several reviews (Lindsay et al., 2015; Linden et al., 2016, 2018; Resch et al., 2018; Wade et al., 2018) suggest that technology-based treatments are a promising approach for the rehabilitation of children with ABI (Corti et al., 2019). Cognitive and behavioral trainings are available (Corti et al., 2019), however the role of the therapist in these programs is usually intermittent and offers scarce opportunities for therapist-patient interaction (Camden et al., 2019). Indeed, in cognitive training the therapist monitors patient motivation, and meetings between the two are scheduled with a weekly frequency to deliver feedback on the performance of the child (Resch et al., 2018). The same happens in the behavioral programs where the majority of the work is performed by the patients and their families alone, and the therapist is present every one or two weeks (Corti et al., 2019). In contrast, the practice of synchronous cognitive training with the presence of the therapist several times a week seems to be under-investigated in literature.

On the other hand, speech therapy conducted via videoconferencing treatments appears to be better suited to the use of real-time telerehabilitation (Theodoros et al., 2008), especially for the improvement of stuttering (Sicotte et al., 2003), voice disorders, laryngectomy, swallowing dysfunction, and speech and language disorders (Grogan-Johnson et al., 2010; Theodoros et al., 2008) in children, or in the treatment of chronic post-stroke aphasia in adult patients (Macoir et al., 2017; 0ra et al., 2020).

Despite the promising results achieved with telerehabilitation, its application in pediatric clinical settings is not yet widespread. There is a lack of studies regarding the feasibility and acceptability of telerehabilitation interventions in clinical settings dedicated to developmental age pathologies (Tanner et al., 2020).

On March 24^th^ 2020, a real-time telerehabilitation intervention program was launched at the Scientific Institute I.R.C.C.S. E. Medea, La Nostra Famiglia, Italy, (hereafter referred to as, “Institute”) with the aim of guaranteeing patients with ABI continuity of care even in the COVID-19 emergency and taking into account the containment and social distancing measures. The intervention was firstly created for clinical purposes and then to evaluate the feasibility and acceptability of a synchronous telerehabilitation intervention during the COVID-19 emergency. The uniqueness of the pandemic could allow a new view of the feasibility and acceptability of a telerehabilitation intervention in an emergency situation, when standard activities have been forced to change. A twofold point of view was provided: not only that of the patients and/or caregivers but also of the therapists. The intervention was designed according to the suggestions offered by renowned associations such as those listed above. Telerehabilitation was delivered with a synchronous modality with the aim of maintaining the close relationship between the therapist and the patient similar to that achieved within the traditional in-person intervention.

## MATERIALS AND METHODS

### PATIENTS

Before the COVID-19 emergency began, 16 patients were undergoing an in-person rehabilitation intervention three times a week for several hours a day, as outpatients. When the pandemic spread, in compliance with the containment and social distancing measures, it was impossible for them to access the Institute. They were offered the possibility of virtually continuing the rehabilitation that they had previously carried out in-person, if they met the following inclusion criteria: (a) they were 5-21 years of age, (b) they had a diagnosis of ABI (namely: brain tumors, traumatic brain injuries, hemorrhagic stroke, haemorrhage, or viral meningoencephalitis, (c) they had cognitive and speech difficulties, resulting from ABI, (d) they were involved in rehabilitation treatments before the spread of COVID-19, (e) they had at least one supportive caregiver (American Psychological Association, 2020; American Telemedicine Association, 2017, 2020; Brennan et al., 2010; Inter Organizational Practice Committee, 2020), and (f) they had a personal computer at home, with a webcam and access to the Internet (American Psychological Association, 2020) to support the use of Google Meet (formerly Hangouts Meet, a free software for real-time meetings, available as an extension for the Google Chrome web browser).

Children and adolescents were excluded if they had: (a) unstable or photosensitive epilepsy (Piovesana et al., 2017), (b) serious behavioral and attention deficits that would have made it impossible to follow the rehabilitation sessions (Wong et al., 2021) (i.e., as assessed with previous standardized tests/questionnaires at admission as standard evaluation of the clinical care), (c) Full Scale Intelligent Quotient (FSIQ) ≤ 55, meaning mild mental impairment (American Psychiatric Association, 2014), or (d) very serious pathologies in comorbidity requiring too many structured treatments which were not suited to telemedicine (Wong et al., 2021).

Out of 16 patients, all met the inclusion criteria and 13 completed the intervention (See session 3 for details). Demographic, clinical characteristics of the participants, and a recent cognitive evaluation, including the FSIQ, the Verbal Comprehension Index (VCI), Visual Spatial Index (VSI), Working Memory (WMI), and Processing Speed Index (PSI) from the age-appropriate Wechsler Intelligence Scales (Wechsler, 1997, 2008, 2012), were extracted from their medical charts (See Table 1). The most frequent diagnosis was brain tumor (23.0%), followed by traumatic brain injury (15.3%). Other diagnoses included hemorrhagic stroke, haemorrhage, or viral meningoencephalitis. More than half of the patients were males (69.5%), mean age at treatment was 10.7 years (128.7 months), and mean time post injury was 2.5 years (30.3 months).

**Table 1:** Patient Clinical Data

	Patients with ABI (N=13)
Age at 24th March 2020 (months):	
M (SD)	128.2 (51.6)
Range	65–244
Gender (N):	
Male	8 (61.5%)
Female	5 (38.4%)
Diagnosis (N/%):	
Traumatic Brain Injury	2 (15.3%)
Brain Tumor	3 (23.0%)
Other	9 (69.2%)
Time from injury to assessment (months):	
M (SD)	23.9 (36.7)
Range	0–134
Time from assessment to 24^th^ March 2020:	
M (SD)	2.8 (2.1)
Range	0–6
Time post injury to 24^th^ March 2020 (months):	
M (SD)	25.9 (36.3)
Range	2–136
Full Scale IQ :	
N	12
M (SD)	92.8 (23.3)
Range	58–129
Verbal Comprehension Index (VCI):	
N	12
M (SD)	97.3 (21.8)
Range	70–134
Visual Spatial Index (VSI):	
N	13
M (SD)	97.7 (23.4)
Range	63–126
Working Memory Index (WMI):	
N	9
M (SD)	87.8 (22.5)
Range	64–121
Processing Speed Index (PSI):	
N	13
M (SD)	84.2 (21.3)
Range	56–112

### INTERVENTION

The intervention was conducted by four experienced therapists: two for neuropsychological sessions (one male and one female) and two for speech sessions (two females). Mean therapist age was 45 years (M=540.7 months; SD=178.1). The therapists had been working for several years at the Institute doing in-person rehabilitation of patients with ABI, and had occasional experience with telemedicine for patients who had difficulty accessing the Institution. The therapists were involved in the present study as questionnaire data collectors.

A preliminary explanation of the project was provided to parents via email and phone calls, to clarify the procedures of the project. Before the study began, specific exercises were selected for treatment of all of the areas of impairment, both for speech and neuropsychological difficulties. Taking into account the general aim of the treatment, each therapist drew up a list of specific goals tailored to each patient's needs; these were shared and agreed upon with the caregivers, and/or older patients if they were able to recognize their own difficulties.

In addition to this “standard” intervention, if a patient showed a specific persistent impairment in a single area, it was at their therapist's discretion to select additional exercises, different from those already proposed. This individualized planning allowed for more personalized interventions for the patients.

Contact with the patient took place individually twice a week, with two real-time sessions of 45 minutes each. After the daily sessions, the therapist encouraged supplementation of the rehabilitation work by providing specific stimulation exercises via email.

The therapy equipment consisted of a personal computer with a 30x38 cm screen, 18.9748 cm, with a resolution of 1280 x 1024, and a webcam with integrated microphone. The symmetrical Internet connection had a speed of 400Mb/s.

The telerehabilitation sessions took place using the free software Google Meet. This allowed the performance of the following real-time working methods:

1. Video call mode (both the therapist and the patient could see one another);
2. Screen sharing mode (which made the session more interactive and more similar to traditional in-person intervention):
  - both shared the patient's screen (in order to monitor user activity),
  - both shared the therapist's screen (the activity was carried out by the operator under the user's guidance),
  - the activity was shared on other devices (i.e., tablet, keyboard, and worksheets) by tilting the video camera.

These modes are used alternately by the therapists according to the objectives selected for the patient. All procedures were documented, shared, and regularly monitored. If a session was missed for a genuine reason (i.e., patient illness, or obligatory caregiver absence) the session could be rescheduled within the next few days to maintain the overall 10-week duration of the intervention.

Parents had signed a standard informed consent form approved by the Ethical Committee of the Institute for in-person rehabilitation and data collection when the patients were admitted to the Institute before the COVID-19 emergency. The only patient included in the sample who was older than 18 years signed his own consent form. This consent was also valid for the provision of real-time telerehabilitation interventions. The study was approved by the Scientific Institute I.R.C.C.S. E. Medea Ethics Committee and was conducted in agreement with the principles expressed in the 1964 Declaration of Helsinki.

There was no extra cost for the patients and their families to participate in the telemedicine project. Indeed, the Health Care System in Italy states that telemedicine interventions are paid for in the same way as interventions provided in-person.

### NEUROPSYCHOLOGICAL TELEREHABILITATION

Neuropsychological treatment focused on the improvement of specific domains, such as attention, visual-constructive skills, mnesic difficulties and executive functions. For details see Table 2.

**Table 2:** Goals of Intervention for Each Function Treated in Neuropsychological and Speech Telerehabilitation

Treatment	Functions	Goals
Neuropsychological Telerehabilitation	Attention	Increase attention span. Pay attention to the task avoiding distracting stimuli.
	Visual-constructive skills	Increase visual and sequential analysis. Correctly identify and use spatial reference points. Increase the capacity of spatial integration.
	Memory	Increase of short-term, long term and procedural memory.
	Executive functions	Increase in task planning, problem solving skills, cognitive flexibility, working memory.
Speech Interventions	Expressive skills	Promoting an expansion of the morpho-syntactic structure. Promoting correct planning. Stimulating the use of logical-causal and temporal links. Expanding semantic-lexical knowledge.
	Pragmatic skills	Improve understanding of indirect speech acts, idiomatic and metaphorical expressions. Reduce tangential derailments in conversational activities by favouring the maintenance of the focus of the speech.
	Phonetic skills	Consolidate emerging phonemes. Favour their generalization in spontaneous speech.
	Phonological skills	Improve phonological programming. Reduce structure and system processes.
	Phonological awareness	Promote the acquisition of metaphonological processes of segmentation and syllabic / phonemic fusion. Improve initial word sound recognition and rhyming recognition.
	Grammatical comprehension	Promote the understanding of specific morphosyntactic structures.
	Lexical-semantic skills	Promote an expansion of semantic-lexical knowledge.

### SPEECH TELEREHABILITATION

Speech telerehabilitation focused on the improvement of several aspects of communication, such as expressive, pragmatic, phonemic, phonological, lexical-semantic skills, and grammatical comprehension. For details see Table 2.

For both neuropsychological and speech interventions, the therapy materials consisted of specific software (mainly by sharing the therapist's screen and verbal indications provided to the child), free online activities (principally with remote therapist control) or tablet apps (if such a device was available to the family).

### MEASURES OF FEASIBILITY AND ACCEPTABILITY

To better define the measurements gathered, an operational definition of the two terms was used to evaluate the telerehabilitation intervention. Based on a previous study (Øra et al., 2020), *feasibility* was defined as the viability of the selected technical features (i.e., audio and image quality and signal reception). Based on the definition proposed by Sekhon et al. (2017), the current study measured *acceptability* both from the therapists' and the caregivers' point of view. The former included satisfaction with the relationship with the patients, achieved goals, compliance, and degree of control over the patient (meaning both engagement of the patients and the therapist's feeling that the patient followed his/her directions). Adherence to the intervention was measured by tracking the patient's attendance at each single session. Acceptability for the patients/caregivers considered satisfaction with the intervention, inclusive of the relationship between the therapist and the patients, and any prior concerns about a remote intervention (Øra et al., 2020).

For this purpose, two survey questionnaires were selected among those available in the literature (Sicotte et al., 2003). The original formats were in English and were translated into Italian. The questionnaires were not validated, but they had already been used in a similar study, with analogous aims (Sicotte et al., 2003). They were administered, one to the therapist (See Table 3) and one to the parents (together with the patients) (See Table 4).

**Table 3:** Questionnaire Completed by the Therapist after Each Session

	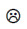				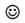
	1	2	3	4	5
1. Sound quality					
2. Signal reception					
3. Image quality					
					
	1	2	3	4	5
4. Quality of the therapeutic relationship with the patient					
5. Degree of control over the patient during treatment					
6. Attainment of clinical goals					
7. Degree of patient's compliance with the instructions given by the therapist					

*Note.* From Sicotte et al., 2003; 1=highly dissatisfied; 3=neutral; 5=highly satisfied

**Table 4:** Questionnaire Completed by the Parents at the End of Telemedicine Program

Not at all satisfied	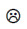		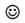
	Not at all satisfied	Somewhat satisfied	Highly satisfied
	**1**	**2**	**3**
1. Are you satisfied with the technical performance (image and sound quality) of the telemedicine treatment?			
2. Are you satisfied with the relationship between the therapist and your son/daughter?			
	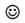		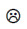
	Not at all concerned	Somewhat concerned	Highly concerned
	**1**	**2**	**3**
3. Were you concerned that your son/daughter was treated from a distance by a therapist?			
	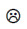		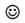
	Not at all useful	Somewhat useful	Highly useful
	**1**	**2**	**3**
4. How useful was the telemedicine treatment for your son / daughter in your opinion?			

*Note*. From Sicotte et al., 2003.

The rating form for therapists used a 5-point Likert scale, where 1=highly dissatisfied, 3=neutral and 5=highly satisfied. The questionnaire was comprised of seven items: the first three evaluated technical aspects, namely sound and image quality, and signal reception (*feasibility*); the final four assessed clinical features, such as quality of the therapeutic relationship with the patient, the degree of control over the patient and the degree of the patient's compliance with the instructions given by the therapist, and finally the attainment of clinical goals *(acceptability).* Each therapist completed the questionnaire independently after each encounter with a patient, without any comparison with colleagues. The unit of analysis in this case was each single session.

A 4-item, 3-point scale questionnaire was administered to parents and assessed the perceived usefulness of the telerehabilitation intervention (1=not at all useful; 3=highly useful), the relationship between the child and the therapist (1=not at all satisfied; 3=highly satisfied) and the concern about the child being engaged in a rehabilitation program from a distance (1= not at all concerned; 3= highly concerned). These three items represented *acceptability.* The item that evaluated any technical problems encountered with sound and image quality (1=not at all satisfied; 3=highly satisfied) indicated *feasibility.* Only one questionnaire was filled in by parents that assessed both neuropsychological and speech interventions at the end of the project. The unit of analysis in this case was the entire intervention. The criterion for intervention success required 18/18 (seven items for the neuropsychological therapists, seven items for the speech-language pathologists and four items for the caregivers) satisfied outcome measures.

### ANALYSIS

Statistics performed using the SPSS 19.0 software were used to describe demographics, clinical variables, and the feasibility and acceptability from the therapists' (See Table 5), and the parents' questionnaires (See Table 6).

**Table 5:** Total Means, SDs, and Range in the Therapists' Questionnaires Assessing Feasibility and Acceptability, for Neuropsychological and Speech Interventions

		M (SD) [Range: min-max]	
		Neuropsychological Therapy	Speech Therapy
**Feasibility**	1. Sound quality	3.9 (0.3) [Range: 3.2–4.4]	4.4 (0.2) [Range: 4.0–4.8]
	2. Signal reception	3.8 (0.3) [Range: 2.9- 4.3]	4.4 (0.2) [Range: 3.7–4.9]
	3. Image quality	4.0 (0.3) [Range: 3.3- 4.4]	4.3 (0.2) [Range: 3.8–4.7]
**Acceptability**	4. Quality of the therapeutic relationship with the patient	3.9 (0.1) [Range: 3.6–4.2]	4.6 (0.2) [Range: 3.9–5.0]
	5. Degree of control over the patient during treatment	3.4 (0.1) [Range: 3.2- 3.7]	4.3 (0.2) [Range: 3.9–5.0]
	6. Attainment of clinical goals	3.7 (0.2) [Range: 3.1–4.0]	4.5 (0.2) [Range: 4.0–5.0]
	7. Degree of patient's compliance with the instructions given by the therapist	3.6 (0.1) [Range: 3.2–3.9]	4.5 (0.1) [Range: 4.2–5.0]

**Table 6:** Items and Scores from the Parents' Questionnaires Assessing Feasibility and Acceptability

	M (SD)
**Feasibility**	
1. Are you satisfied with the technical performance (image and sound quality) of the telemedicine treatment?	2.6 (0.6)
**Acceptability**	
2. Are you satisfied with the relationship between the therapist and your son/daughter?	3.0 (0)
3. Were you concerned that your son/daughter was treated from a distance by a therapist?	1.0 (0.2)
4. How useful was the telemedicine treatment for your son/daughter in your opinion?	2.9 (0.2)

## RESULTS

**Figure 1:**
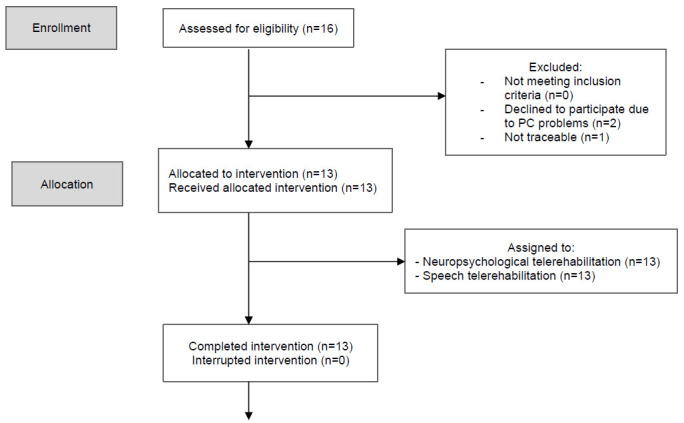

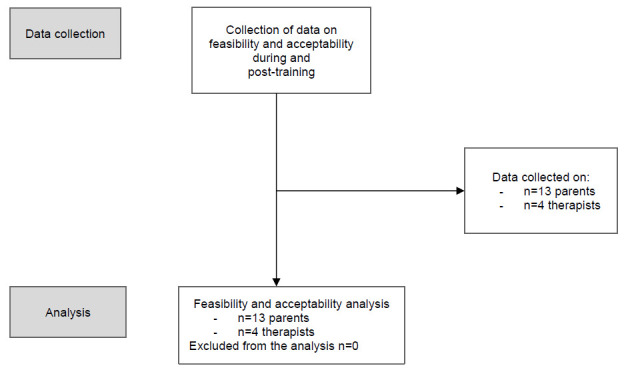
CONSORT Flowchart of Participant Enrollment, Allocation, Data Collection and Analysis

Figure 1 shows the different steps of the interventions and their evolution over time. Sixteen patients were asked to participate: 81.2 % accepted the proposal to take part in the project. Two families refused to participate because of technical problems with their personal computers, and one patient was no longer traceable. Thirteen children were allocated to and started the interventions. All of them completed the 20 sessions and only five meetings were missed but then rescheduled, with three different patients. Data were collected on 13 families and four therapists and all were used for the analysis. No modification was applied to the intervention during the project.

### PATIENT CHARACTERISTICS

For one patient only the VSI was available because he had expressive language difficulties (for him this index, which is > 55, was considered valid, as inclusion criteria). The average FSIQ for the remaining patients was 92.8 (SD=23.9), thus in the normal range, like the VCI (M=97.3; SD=21.8) and VSI (M=97.7; SD=23.4), while the PSI fell in the low average range (M= 84.2; SD=21.3) like WMI (M=87.8; SD=22.5). This latter index was available for nine patients (69.2%) out of 13 because three were evaluated with the Wechsler Preschool and Primary Scale of Intelligence-3^rd^ Edition, and with the Wechsler Adult Intelligence Scale-Revised, which do not include this score (See Table 1). According to the assessment performed at admission, all patients exhibited some type of impairment, both in neuropsychological and communication functioning. The majority displayed more than one impaired neuropsychological or communication domain. The number of hospitalizations the patients had before the COVID-19 emergency was noted (excluding the one in 2020 that was then switched to a remote modality). Two patients did not undergo any previous rehabilitation stay. Six patients underwent just one hospitalization before 2020. Two patients were on their third stay and two on their fourth. Finally, just one patient had a long history of rehabilitation, with ten hospitalizations at the Institute.

### THERAPIST FEASIBILITY AND ACCEPTABILITY OUTCOME

The criterion for intervention success was achieved: 14/14 was obtained for therapist satisfaction outcome measures, considering the overall means, for both interventions. Overall feasibility and acceptability outcomes drawn from both the neuropsychological therapists and speech-language pathologists are presented in Table 5.

#### NEUROPSYCHOLOGICAL THERAPY SESSIONS

Two neuropsychological therapists with 20 sessions for each patient completed a total of 98.4% questionnaires. Only four session questionnaires (1.5%) were partially filled in because technical problems on that particular day did not permit the evaluation of the clinical items.

Apart from these isolated difficulties, no significant technical issue was found, and technical quality was judged by the therapists as being very good. Overall, on the five-point scale 48.3% of the sessions were rated as 3 and 51.6% as 4 or more for technical quality.

*Feasibility:* All 3 criteria were met. Image quality (Item 3) was rated with the highest score, both for technical quality and among all the 7 items (M=4.0; SD=0.3), while the lowest score was for signal reception (Item 2: M=3.8; SD=0.3).

*Acceptability:* All 4 criteria were met. Generally, a rating of 3 was given for 85% of the sessions and 15% were given 4 or more for acceptability. The highest score for neuropsychological therapy acceptability was obtained for the quality of the therapeutic relationship with the patient (Item 4: M=3.9; SD=0.1), while the degree of control over the child during the treatment was scored with the lowest result (Item 5: M=3.4; SD=0.1). See Table 5 and Figure 2.

**Figure 2a:**
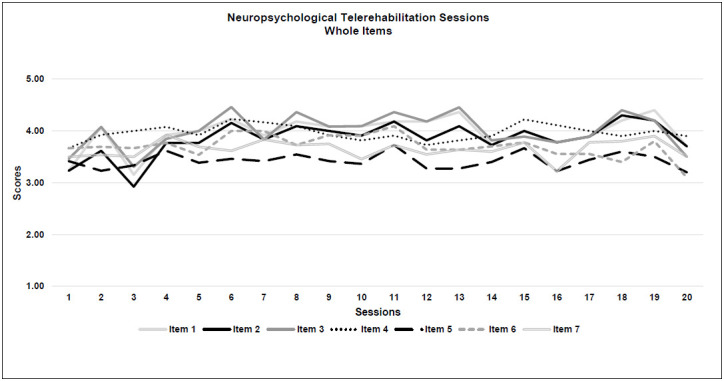
Neuropsychological Telerehabilitation Sessions (Whole Items)

*Note.* Mean scores of the neuropsychological therapists' questionnaires: (a) whole items, (b) feasibility, and (c) acceptability of neuropsychological therapy throughout the 20 sessions. Item 1: Sound quality; Item 2: Signal reception; Item 3: Image quality; Item 4: Quality of the therapeutic relationship with the patient; Item 5: Degree of control over the patient during treatment; Item 6: Attainment of clinical goals; Item 7: Degree of patient's compliance with the instructions given by the therapist.

**Figure 2b:**
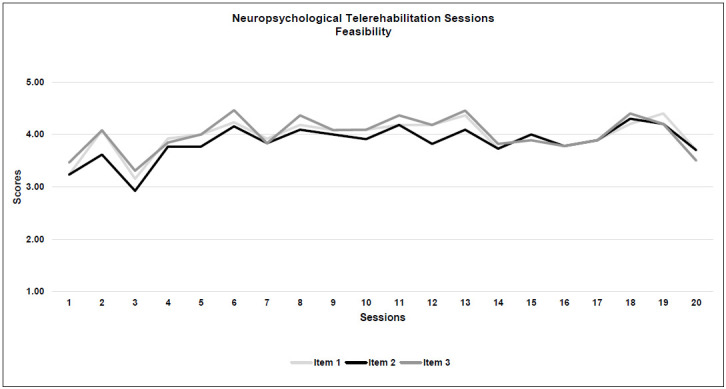
Neuropsychological Telerehabilitation Sessions (Feasibility)

*Note.* Mean scores of the neuropsychological therapists' questionnaires: (a) whole items, (b) feasibility, and (c) acceptability of neuropsychological therapy throughout the 20 sessions. Item 1: Sound quality; Item 2: Signal reception; Item 3: Image quality; Item 4: Quality of the therapeutic relationship with the patient; Item 5: Degree of control over the patient during treatment; Item 6: Attainment of clinical goals; Item 7: Degree of patient's compliance with the instructions given by the therapist.

**Figure 2c:**
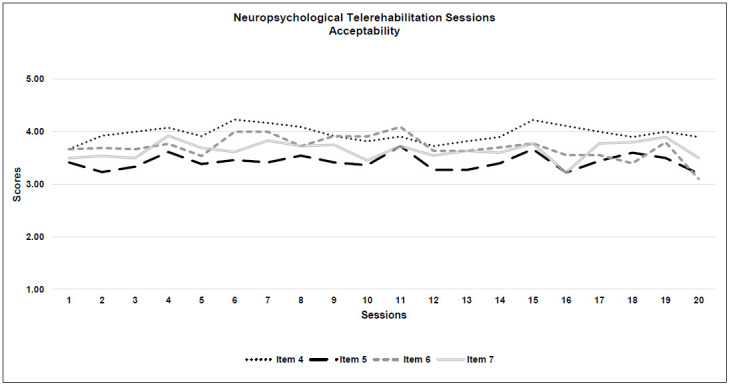
Neuropsychological Telerehabilitation Sessions (Acceptability)

*Note.* Mean scores of the neuropsychological therapists' questionnaires: (a) whole items, (b) feasibility, and (c) acceptability of neuropsychological therapy throughout the 20 sessions. Item 1: Sound quality; Item 2: Signal reception; Item 3: Image quality; Item 4: Quality of the therapeutic relationship with the patient; Item 5: Degree of control over the patient during treatment; Item 6: Attainment of clinical goals; Item 7: Degree of patient's compliance with the instructions given by the therapist.

#### SPEECH THERAPY SESSIONS

Two speech-language pathologists with 20 patients each completed all the questionnaires. No significant technical issue was found and 96.6% of the session ratings were of 4 or more for technical quality.

*Feasibility:* All three criteria were met. Sound and signal quality during the speech sessions were both rated with the highest score (Item 1: M=4.4; SD=0.2; Item 2: M=4.4; SD=0.2), while the remaining technical item (no. 3, Image quality) was the lowest (M=4.3; SD=0.2).

*Acceptability:* All four criteria were met. Overall, almost all of the sessions (97.5%) were rated 4 or more for clinical quality. As for the technical part, the quality of the therapeutic relationship was given the highest score for clinical quality and also among all the 7 items (Item 4: M=4.6; SD=0.2), while the degree of control over the patient had the lowest outcome (Item 5: M=4.3, SD=0.2). See Table 5 and Figure 3.

**Figure 3a:**
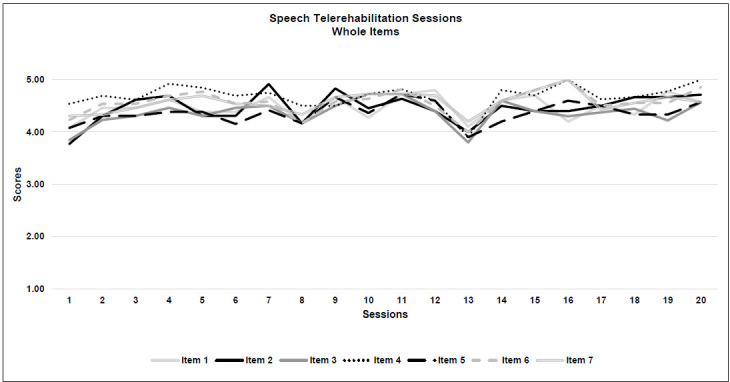
Speech Telerehabilitation Sessions (Whole Items)

*Note.* Figure 3: Mean scores of the speech-language pathologists' questionnaires: (a) whole items, (b) feasibility and (c) acceptability of speech therapy throughout the 20 sessions. Item 1: Sound quality; Item 2: Signal reception; Item 3: Image quality; Item 4: Quality of the therapeutic relationship with the patient; Item 5: Degree of control over the patient during treatment; Item 6: Attainment of clinical goals; Item 7: Degree of patient's compliance with the instructions given by the therapist.

**Figure 3b:**
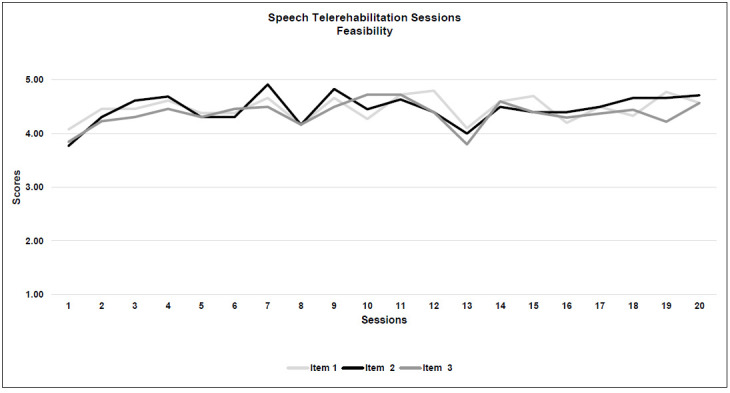
Speech Telerehabilitation Session (Feasibility)

*Note.* Figure 3: Mean scores of the speech-language pathologists' questionnaires: (a) whole items, (b) feasibility and (c) acceptability of speech therapy throughout the 20 sessions. Item 1: Sound quality; Item 2: Signal reception; Item 3: Image quality; Item 4: Quality of the therapeutic relationship with the patient; Item 5: Degree of control over the patient during treatment; Item 6: Attainment of clinical goals; Item 7: Degree of patient's compliance with the instructions given by the therapist.

**Figure 3c:**
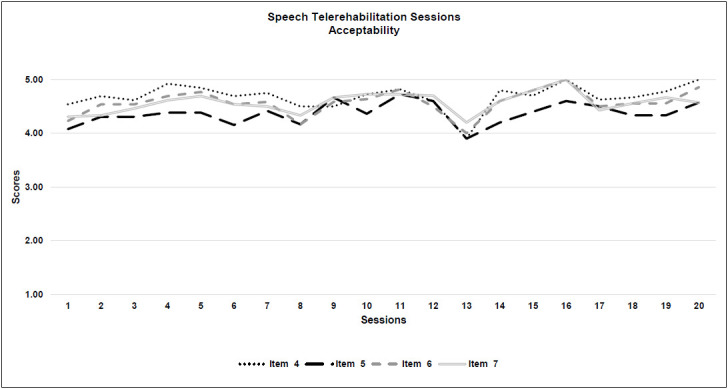
Speech Telerehabilitation Sessions (Acceptability)

*Note.* Figure 3: Mean scores of the speech-language pathologists' questionnaires: (a) whole items, (b) feasibility and (c) acceptability of speech therapy throughout the 20 sessions. Item 1: Sound quality; Item 2: Signal reception; Item 3: Image quality; Item 4: Quality of the therapeutic relationship with the patient; Item 5: Degree of control over the patient during treatment; Item 6: Attainment of clinical goals; Item 7: Degree of patient's compliance with the instructions given by the therapist.

#### PARENT FEASIBILITY AND ACCEPTABILITY

**Figure 4:**
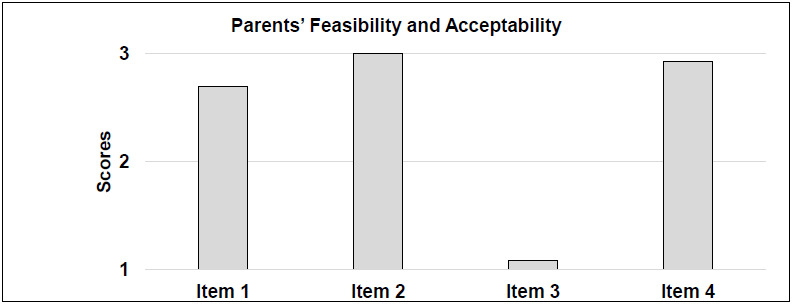
Parents' Questionnaire Scores: Feasibility and Acceptability of Overall Telemedicine Intervention

The criterion for intervention success was achieved: 4/4 was obtained for caregiver satisfaction outcome measures, considering the overall means. Parent questionnaire results are shown in Table 6 and Figure 4.

*Feasibility:* For Item 1 (Technical quality of sound and image) the majority of the parents rated 3 (N=10, 76.9%), two parents rated 2 (15.3%) and only one rated 1 (7.6%) because they had had several problems with their Wi-Fi connection.

*Acceptability:* Item 2 was about the clinical relationship between patients and therapist: all assigned a score equal to 3. The majority of caregivers were not worried about the telemedicine intervention before starting, indeed they gave a score of 1 on Item 3 (N=10, 76.9%). Two caregivers (15.3%) were just a little worried about treatment at a distance. Only one parent (7.6%) was very anxious before the beginning of the intervention. Item 4 evaluated the general usefulness of the telemedicine intervention: 12 caregivers (92.3%) rated 3, only one (7.6%) gave 2, and from a qualitative point of view most parents reported that the intervention was very beneficial and that they were very relieved not to waste the months of lockdown without the intervention of a therapist.

#### CAREGIVER SUPPORT

Therapists recorded the possible presence of a parent in each session. Two patients (who were twins) required the presence of their mother during all sessions (n = 40), both for neuropsychological and speech therapy.

Two patients, both 5 years old, required their caregivers' presence during almost all the interventions (n = 36 and n = 34). One of those patients required their caregivers' presence for 19 sessions for neuropsychological therapy and 17 for speech therapy, probably due to his intellectual functioning (FSIQ = 71). The other patient required caregiver support for 14 sessions for neuropsychological therapy and 20 for speech therapy (due to a fine-motor impairment).

Four patients were 7 years old or older. Two of the four needed their caregivers in six sessions during neuropsychological therapy, but for speech therapy the caregiver was present for 11 and 19 sessions each, (for a total of 17 and 25 sessions respectively) because both had more difficulties with speech than with neuropsychological functioning. The other two patients required the caregiver only in three sessions, all for neuropsychological teletherapy and none for speech teletherapy.

Two patients needed caregivers for the speech teletherapy for one and two sessions respectively, with none needed for neuropsychological teletherapy. One patient needed two sessions with caregivers, both for neuropsychological and speech therapy (n = 4). The remaining two patients were completely autonomous in all the sessions.

**Table 7:** Caregiver Presence for Neuropsychological, Speech Telerehabilitation and Overall Sessions for Each Patient

	Caregiver presence		
Patient	Neuropsychological telerehabilitation	Speech telerehabilitation	TOT.
1	20	20	40
2	20	20	40
3	0	2	2
4	3	0	3
5	6	19	25
6	6	11	17
7	2	2	4
8	3	0	3
9	0	0	0
10	19	17	36
11	14	20	34
12	0	0	0
13	0	1	1

## DISCUSSION

The goal of the study was to test the feasibility and acceptability of a real-time telerehabilitation intervention for a population of paediatric patients with ABI, during the emergency caused by COVID-19.

With respect to feasibility and acceptability, therapist questionnaires reported no interfering issues that limited the delivery of the treatments, thus demonstrating the suitability of the intervention. The questionnaire scores were stable throughout the 10-week period and remained at a high average level, meaning that the interventions were positively managed. This was not a foregone conclusion. The scores could have changed; for instance, the measures could have improved after the first few sessions after the therapists and the patients adapted to the new virtual interaction. On the other hand, the scores may have decreased, if the therapists and the patients tired from meeting virtually. Moreover, neither the patients nor their families experienced significant technical or clinical issues; on the contrary, they were very satisfied with the intervention. Patients were positively engaged by this new method of treatment provision, suggesting that real-time telerehabilitation could be an appropriate proposal for paediatric patients with ABI.

A more precise analysis of the seven individual items leads us to some important considerations about real-time telerehabilitation.

### FEASIBILITY

As far as feasibility is concerned, the evaluation of the three items differed in the two interventions: for neuropsychological treatment image quality had the highest score, while signal reception and sound quality were the highest in speech therapy (even though the differences between the scores were a matter of decimals). This is interesting because usually neuropsychological interventions are based on a massive use of images, work sheets, and other visual aids, while speech therapies are often more focused on sounds.

### ACCEPTABILITY

With regard to acceptability, the quality of the therapeutic relationship was the item that obtained the highest score in both types of treatment. In current literature, while several studies report the importance of the therapeutic relationship in in-person interventions, the role of this same factor in telerehabilitation is unsettled. A review by Sucala et al. (2021) suggested that the therapeutic alliance in these two types of intervention is similar. That is in line with the current results because the patients had already met their therapists in-person before the pandemic, although not for very long. This constitutes a hybrid condition; conclusions on this aspect should therefore be made with caution and should not be generalised to other situations (i.e., telerehabilitation with no previous in-person meeting) without further research. It is noteworthy that the pre-existing relationship was a very useful link in the transition between the in-person treatments and the remote ones when the emergency spread. At the same time, this issue raises an important question about the utility of a previous in-person meeting when the telerehabilitation is delivered to a paediatric population. To our knowledge, this issue has not yet been investigated. It is of note that two patients in the current sample had no history of any kind of rehabilitation, because their injuries occurred just a few weeks before the pandemic. Although this is too small a number to draw any conclusions, it is encouraging that they did not encounter any difficulties in participating in the intervention remotely.

Several other factors had probably come into play to create a good relationship, such as the expertise of the therapists, the tailored program drawn up for each patient, the usefulness of the program, and the children's condition of pan demic caused isolation. As regards the latter factor, stay-at-home measures contributed to removing some of the classic factors that led patients and parents to refuse to participate in telerehabilitation at home, that is, the time-consuming nature of the intervention causing absence from school and other social activities.

The acceptability item with the lowest score was the degree of control over the patient for both types of interventions. This is a significant point to consider, even though the scores were high overall. Therapists reported that the obvious physical distance from the patients made it difficult to manage certain activities with them, (i.e., to organize work with patients who are constantly moving around, or to engage reluctant children). Therefore, it was necessary to develop and implement a strategy based on verbal interaction (Resch et al., 2018) to reduce the moments of silence between therapist and the child and to engage the attention of the patient, using a more direct technique. This made the session more demanding from the therapist's point of view because of the great number of required verbal interventions that otherwise were not as necessary for in-person treatment. Moreover, the synchronous telerehabilitation therapy required the session to have a more specific structure, with preparation of the necessary materials in advance. Therefore, this intervention was more challenging for the therapist, especially when dealing with younger children. These results seem to be in accordance with those found by Sicotte et al. (2003).

### WHOLE ITEMS OVERVIEW

Considering the overall trajectories of both types of intervention, for the neuropsychological therapists' questionnaires, th e item pattern was more spread out and between a score range of 3 and 4, while the trajectory for speech therapy was more compact, in a score range between 4 and 5. That is consistent with current literature, because speech therapy seems to fit better with the implementation of real-time telerehabilitation (Theodoros et al., 2008). Interestingly, speech-language pathologist questionnaires showed a deviation in all the 7 item scores around session 13, perhaps due to the effects of fatigue, even though the scores remained high. For most of the patients, the 13^th^ session coincided with the beginning of the month of May 2020, during which the lockdown in Italy was easing.

### DIFFICULTIES WITH TELEREHABILITATION

The main obstacle that the neuropsychological therapists encountered was related to the patient's impairment in the executive function domain: if abilities such as planning, monitoring or selective attention were seriously lacking, it was not infrequent that the child was not able to work independently. In these cases, the caregiver's support was necessary to help the child to stay focused, to plan the different steps to complete a task, and to follow them until the end of the activity.

Although telemedicine seems to be suitable for the treatment of speech disorders because interaction occurs for the most part on an auditory and visual level (Cherney et al., 2012), working on receptive language skills turned out to be more complex, especially for the auditory attention (i.e., identification of sounds/noises) and phonological discrimination of minimal pairs of syllables and words.

Overall, the selected characteristics of the real-time telerehabilitation intervention seemed suitable for this kind of population during the COVID-19 emergency.

### SYNCHRONICITY

Real-time telerehabilitation can bring together the best aspects of in-person interventions with a remote telemedicine approach, maintaining the close contact between the patients and the therapist of the former, and the flexibility of the distance of the latter. A review of the effective intervention characteristics for children with disability suggested that real-time interventions delivered directly to the children were associated with a high percentage of improvement in their outcomes (Camden et al., 2019). Although this study is not about the efficacy of the current intervention, it is important to have proof supporting one of the main characteristics selected and find it associated with greater improvements.

Synchronous intervention demonstrated several advantages. First, the constant contact with the therapist contributed to keeping motivation high (Camden et al., 2019) and the patient accepting novel types of treatment. Among the barriers to telemedicine cited in the relevant literature, skepticism about technology was mentioned (Negrini et al., 2020b). Before the beginning of the project this was also a great concern for the telerehabilitation team, but in this time of great stress both patients and their parents were positive about the proposal of a real-time treatment from a distance; they were highly motivated to continue the routine rehabilitation.

Second, patients with ABI are often diagnosed in comorbidity with depression or emotional disorders (Hendry et al., 2020). In the current experience, parents reported that the relationship with the therapist contributed to supporting the child in facing an extremely critical period, with positive effects on mood and energy level.

Third, the therapist functioned as a daily link between the Service and the patient, to constantly monitor the child's condition and the progress of the telerehabilitation activity. This allowed the professionals to identify at-risk conditions, permitting early interventions.

Finally, in Italy, parents and patients perceived online schooling as being somewhat lacking, with less structured educational activities, especially for younger students, leaving the children unoccupied for several hours per day. On the other hand, they reported the advantageous effects of being almost daily engaged in a useful activity such as real-time telerehabilitation.

### PATIENT-CENTERED APPROACH

Some studies seem to suggest that a parent coaching approach is more effective than a patient-centered one for telerehabilitation delivered to children with disabilities, but these studies aimed at improving the patient's behavior or their motor difficulties (Camden et al., 2019). Perhaps that type of intervention is more useful with children with low cognitive functioning or serious physical illness. In contrast, the current sample was composed of children with at most mild mental delay, rather than motor impairment, who were capable of managing a telerehabilitation program.

Moreover, the current intervention was focused on the patient and was not family-centered, to ease the burden of the caregivers. Parents did however have an active role as supervisors while the treatment was taking place. Yet, the ultimate responsibility for the therapy was assumed by the therapist. This helped parents to reduce their stress and the perception of a low self-efficacy. Indeed, no parent reported any difficulty or excessive effort in supervising their children during synchronous telerehabilitation activities. A further positive matter is that parents could see their children's therapy sessions, their progress (Koterba et al., 2020) and how the therapist handled some problems that arose due to the ABI condition.

### AIMING AT IMPROVING SPECIFIC ABILITIES

The selection of the impaired domain(s) on which to intervene and the consequent tailored program built for the child's needs is a key factor for the success of the telerehabilitation intervention and for the caregiver's (and patient's) satisfaction. Moreover, previous research on patients with ABI in the developmental age reported the lack of cognitive intervention for populations with IQ < 70 (Corti et al., 2020; Johansson et al., 2012). Therefore, the feasibility and acceptability results suggest that this kind of intervention is suitable precisely for the children who need it most, patients with mild disability.

### MULTI-WEEKLY FREQUENCY AND 10-WEEK DURATION

The therapeutic relationship and the constant contact that patients and families had with the therapists was decisive in maintaining the progress of the intervention. Moreover, the intensity of the telerehabilitation intervention is reported in literature as being a key factor in maintaining patient motivation (Camden et al., 2019; Palisano et al., 2019). The majority of the cognitive or behavioral interventions for children with ABI required more than 1 day per week of asynchronous commitment, but the real-time contact with the therapist ranged from none, to once a week or to just once every 2-4 weeks (Corti et al., 2019). A total of 20 sessions of training were carried out by participants and 100% of compliance with the project was certainly very encouraging. Literature about cognitive and behavioral intervention for patients with ABI at a developmental age reported a similar duration, ranging from 5 to 20 weeks for the former (with an asynchronous engagement varying from 2 to 6 days per week) to 4-10 weeks for the latter (Corti et al., 2019).

The mean number of sessions for the speech telerehabilitation also ranged from 12 to 20 (Grogan-Johnson et al., 2010; Sicotte et al., 2003). It is of note that the relevant literature suggested that online scheduled encounters seemed to be more useful, than a needs-based approach (Camden et al., 2018).

### TELEMEDICINE: TOWARD A STANDARD OF CARE?

During the COVID-19 emergency, a spontaneous question arose: Could telerehabilitation become a standard of care? Usually, telerehabilitation interventions are complementary to traditional in-person care (Kloek et al., 2019; Koppenaal et al., 2020), but they have several advantages that could lead them to becoming part of the medical routine, replacing, in some cases, traditional in-person intervention.

Telerehabilitation could have a great impact on sustainability: it has the potential to reach a greater number of patients, directly in their own homes, limiting their family journeys and costs to reach the rehabilitation facilities as well as reducing the overall financial burden (i.e., allowing parents to keep on working at their jobs). Moreover, thanks to its versatile nature, telerehabilitation could be applied to several other contexts, both in terms of different clinical populations and for clinical purposes.

## LIMITATIONS

There are some limitations to this study. First, unfortunately two families refused to participate because of technical problems with their personal computers. Telerehabilitation must necessarily be supported by the adequate equipment and Wi-Fi connection. Institutions should do their utmost to help families in need, for example by lending appropriate devices, when possible.

A second important limitation was related to a potential sex bias: the majority of the caregivers that completed the questionnaires were mothers (11/13; 84.6%). Since the literature has reported a “rater bias,” in which mothers and fathers provide different assessments of the same behaviour of their child (van der Valk et al., 2001), it is possible that fathers could have rated the questionnaires differently than mothers. Moreover, of the four therapists, three were female and therefore a similar bias related to the sex could be present in their evaluations. Further studies are needed to better examine this topic.

Generalizability of the work, in terms of external validity, could be limited by several factors such as the small number of patients the project was offered to and the variety of their aetiologies. This could have obscured difficulties present in a specific group of patients. Including a higher number of participants with a specific diagnosis in a future project could be useful to further examine the implications of specific aetiologies in telemedicine rehabilitation. Other limiting factors include the strict selection criteria applied that clearly excluded, at least in this case, a great number of patients.

Additionally, because patients in the current sample did not show any motor impairments it was more useful to include only the neuropsychological and speech therapy treatments in the telerehabilitation project, and no physical therapy. However, telerehabilitation has also been used to improve physical disorders in children with ABI (Grogan-Johnson et al., 2010), so exploring this issue could be useful.

As a feasibility and acceptability study, the present paper does not report the possible improvement in patient abilities after the telerehabilitation interventions. This should be addressed in future studies. Moreover, the therapists' questionnai res were rated on a 5-point Likert scale, while that of the parents was on a 3-point Likert scale. Giving caregivers more choices to evaluate the intervention may be more helpful in better understanding the role of telerehabilitation, along with the possibility of having two separate questionnaires, one for patients and one for parents.

Finally, the COVID-19 emergency was a unique condition, free from the usual factors that could limit participation in these kinds of interventions, such as school or extra-school activities or parent commitments.

## CONCLUSIONS

This study, conducted during the COVID-19 emergency, demonstrated the feasibility and acceptability of synchronous telerehabilitation treatment for young patients with ABI and cognitive and speech difficulties. The results demonstrated that telerehabilitation can be a successful intervention for this population.

Taking into account the positive response of patients to this kind of treatment and the feelings of their parents about not being abandoned by clinicians, it would be helpful to evaluate the implementation of other varied telerehabilitation proposals. Further studies are needed to make telerehabilitation an integral part of the standard of care.

## Data Availability

The data used to support the findings of this study are available from the corresponding author upon request.
